# Practical Points

**Published:** 1892-04-09

**Authors:** 


### PRACTICAL POINTS.

*Ward Walls.—Where painting of ward walls is adopted, how often should the coat be renewed, and in what manner should the walls and ceiling be cleaned in the interim?*

The period during which a painted surface will last depends upon the atmosphere to which it is exposed. In large towns where the air is laden with smoke and other impurities the period will be shorter than in the country. As a general rule, if the paint is well done in the first instance, and protected by two coats of good varnish, it should not need renewal in less than 5 years under the most adverse circumstances. During the interim, the paintwork should be washed at least once a year, and injured places or cracks repaired. The ceiling, if distempered, should be cleaned down to the bare plaster and re-distempered. The important point to secure is that the size used is the very best, and absolutely fresh.

